# Autologous leaflet reconstruction for aortic valve endocarditis

**DOI:** 10.1016/j.xjtc.2025.04.016

**Published:** 2025-05-03

**Authors:** Timothy W. James, J. Hunter Mehaffey, Vinay Badhwar, J. Scott Rankin

**Affiliations:** a Department of Cardiac Surgery, St Joseph's Medical Center, Tacoma, Wash; b Department of Cardiovascular and Thoracic Surgery, West Virginia University, Morgantown, WVa

**Figure:**
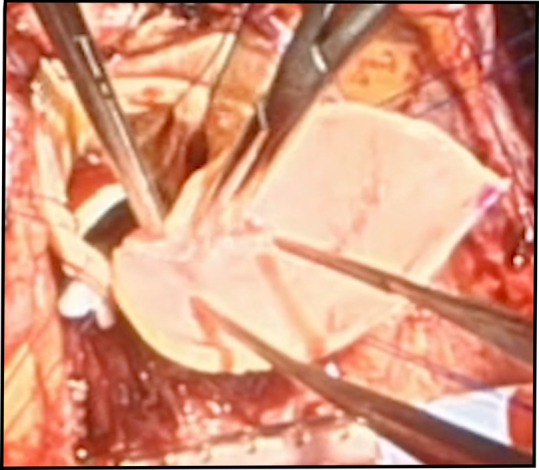
Suturing a leaflet of autologous aortic wall to the right annulus for endocarditis repair.

**Central Message**

A patient with aortic valve endocarditis had leaflet defects reconstructed using autologous aortic wall. Results were excellent. Aortic wall leaflet repair could improve management of endocarditis.

Efforts to repair aortic valves damaged by infective endocarditis (IE) have yielded suboptimal results.^1^^,^^2^ In a representative series of 100 patients with active IE, only one third could be repaired, and in patients with a successful repair, only two thirds were free of grade II or worse aortic insufficiency (AI) at 5 years. Thus, just approximately 20% of overall IE cases could achieve a stable long-term repair. AI recurrence and reoperation occurred more frequently with bicuspid disease and when large patches were required. Because glutaraldehyde-fixed pericardium frequently was used to replace damaged leaflet tissue, late failure from pericardial degeneration was common. Similar to the mitral valve,^3^ a suggestion existed that survival was better with repair for IE, as compared with replacement, but excessive repair failure rates with pericardial patches^4^ precluded a recommendation for routine repair.

A possible solution to this problem is using living autologous aortic wall as an aortic leaflet substitute.^5^^,^E1, E2, E3, E4 The rationale is that autologous aortic wall (1) can receive significant diffusion-related oxygenation and nutrition from both the intimal and adventitial surfaces^E5^ and should remain viable after implantation; (2) is accustomed to systemic pressure and should maintain its structure; (3) is easy to handle and holds sutures well; (4) is readily available in most patients; and (5) is replaceable with simple Dacron grafts. Thus, living aortic wall functioning as leaflet material potentially could achieve long-term biological viability and stable performance after leaflet replacement. Out to a maximal 3 years of follow-up, results with aortic wall patches have been excellent.^E4^ Previously, aortic wall patches have been used in IE only for leaflet perforations, but IE often destroys entire leaflets, necessitating complete leaflet replacement. This Case Video documents a method for replacing entire leaflets with aortic wall patches.

## Methods and Results

### Development of the Leaflet Model

**Figure 1:**
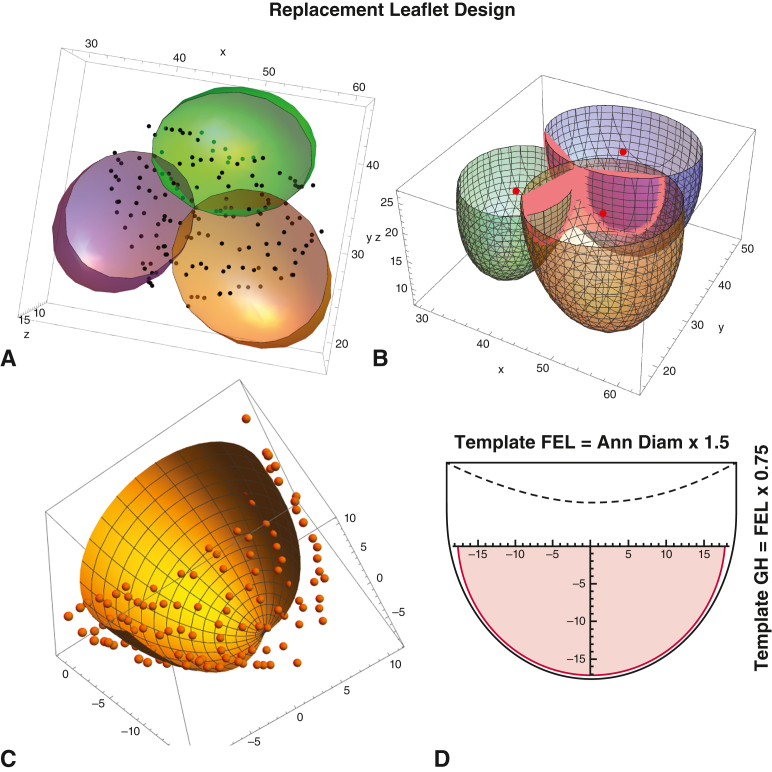
Techniques of analysis used to design replacement leaflets. A, Raw 3-dimensional data points for all 3 leaflets. B, Leaflet geometry defined by ellipsoidal regression with coaptation zones removed. C, Single leaflet data points compared to regression geometry. D, Development of 2-dimensional single leaflet templates from emperic data. See text for details. *FEL*, Free-edge length; *GH*, geometric height.

In a study of aortic valve and root geometry in healthy, awake humans,^E6^ the 3-dimensional coordinates of all root structures were defined manually with high-resolution computed tomography angiography. This process allowed the design of internal annuloplasty rings for repair of both trileaflet and bicuspid valves.^E7^ As part of the data set, however, 3-dimensional coordinates also were available for the leaflets, allowing the current analysis of precise leaflet geometry. In Figure 1, *A*, the 3 leaflet-sinus complexes (as generated by 3-dimensional ellipsoidal regression analyses) are illustrated to the top of central leaflet coaptation, with all extra-leaflet data points cut away. Notice that over the areas of coaptation, the leaflets were compressed, and data points fell between the ellipsoids. In Figure 1, *B*, leaflet data for the coaptation region were removed manually, deleting approximately 25% of leaflet data points. However, enough data still were available (Figure 1, *C*) to generate an accurate picture of the whole leaflet by ellipsoidal regression analysis excluding the compression of coaptation. The modelled leaflet, to the top of central coaptation, then was laid down flat—as shown in the *pink area* in Figure 1, *D*.

**Figure 2:**
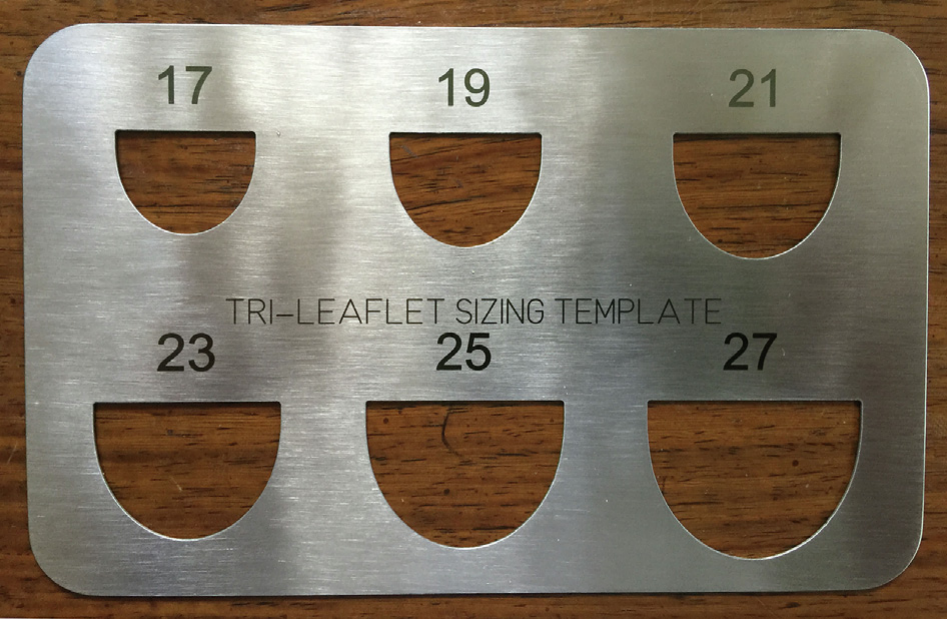
Surgical stainless-steel leaflet templates used to construct neoleaflets of aortic wall for annular/leaflet sizes 17 to 27 mm. Templates were fabricated by Centex Machining, Inc.

In the analysis, annular diameter (AD) was calculated as basal valve circumference/π. The free-edge length (FEL) of the leaflet at the top of central coaptation routinely approximated AD × 1.5 (π/2), confirming previous human cadaver studies,^E8^ with minimal variability in the normal valve.^E5^ The vertical leaflet geometric height to the central top approximated FEL × 0.5, consistent with a hemispherical leaflet model.^E6^ Of course, the commissures always coursed much greater up the aorta and were variable in height. Therefore, in the final neoleaflet template (*black line* in Figure 1, *D*), an additional upper segment of leaflet was provided (0.25 × FEL) to allow for matching the leaflet to the commissural tops and for trimming the central neoleaflet to, or just above, the level of the native leaflets (*dashed line*). Multiple engineering models were evaluated to represent the shape of the neoleaflet, and the best was a spline of a linear segment above and a parabola below (shown in Figure 1, *D*). A view of the completed leaflet templates constructed from surgical stainless steel is provided in Figure 2. In the final template, the numbers represent the ideal valve diameters for each leaflet FEL (again AD = FEL/1.5). Typically, the FEL of a normal leaflet would be measured with a ball sizer (#301; Corcym SRI) (Video 1) to define the ideal diameter (and therefore proper leaflet coaptation) for a given valve.^E7^ If AD were 2 or more sizes larger that leaflet FEL would predict, and appropriate annuloplasty ring would be placed.

### Informed Consent

It has been determined previously^5^ that aortic wall patch procedures primarily pose an issue of informed consent, and therefore, detailed informed consent was provided preoperatively, including theoretical advantages of autologous tissue, as well as limited follow-up data available. Written consent also was acquired for publication of clinical data and video images. Finally, institutional review board approval was obtained (WVU# 2005016064; approval date May 29, 2020; expiration date May 28, 2025). Deidentified videos, associated echocardiograms, and patient demographic data subsequently were assembled and produced.

### Clinical Presentation

**Figure 3:**
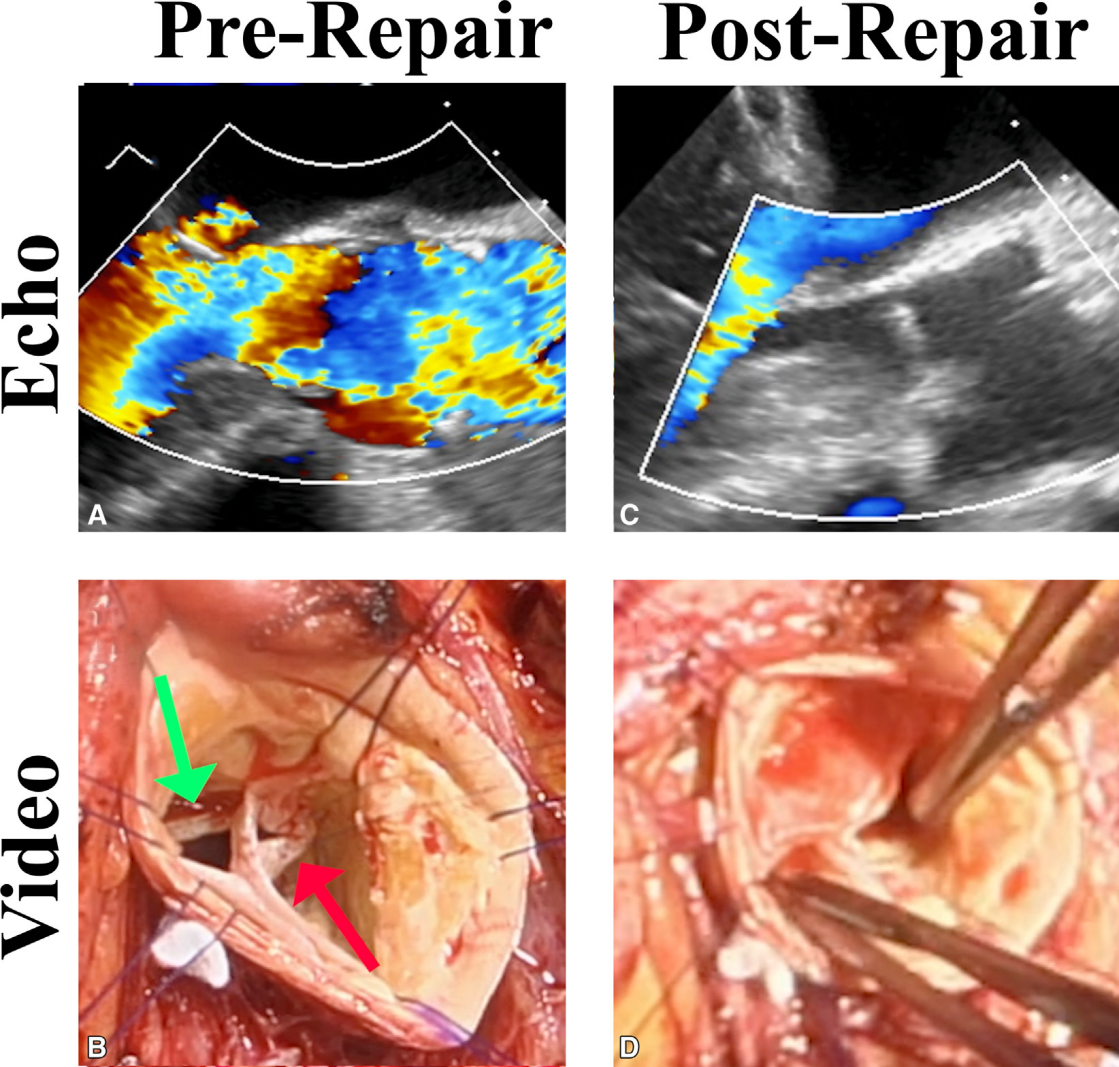
A, Prerepair echocardiogram showing severe AI. B, Prerepair pathology with a perforation in the noncoronary leaflet (*red arrow*) and an absent right coronary leaflet (*green arrow*). C, Postrepair echocardiogram showing no AI. *D*, Good matching and coaptation of the neoright coronary leaflet of aortic wall with the other native leaflets. *AI*, Aortic insufficiency.

The patient was a 66-year-old woman admitted with class IV heart failure, respiratory distress, and bronchitis/pneumonia. A murmur was noted, a transesophageal echocardiogram showed severe AI with aortic leaflet vegetations, and *Enterococcus faecalis* was cultured from her blood. The right coronary leaflet prolapsed with an eccentric posteriorly directed AI jet (Figure 3). Left ventricular function and coronary anatomy were normal. She was treated with a full course of intravenous vancomycin and medical therapy for her heart failure. Pulmonary function tests were normal. Preoperative computed tomography scanning showed a normal ascending aorta, blood cultures converted to negative, but she remained symptomatic at class III.

### Operative Management

At surgery (Video 1), the right coronary leaflet was found to be destroyed with only a crescent shaped rim remaining. The noncoronary leaflet had a 4 × 6-mm perforation near the right-non-coronary commissure (Figure 3). The annulus was normal and measured 21 mm with a Hegar dilator. Ball sizers measured left- and noncoronary leaflets at 19 mm. Because the annulus was essentially normal, annuloplasty was not performed. The ascending aorta was harvested, trimmed of adventitial fat, and then used as leaflet replacement material.

The leaflet perforation was closed radially to avoid any loss of geometric height, using fine 7-0 PROLENE sutures placed horizontally through either side of the leaflet perforation, then through a single 2 × 4-mm strip of aortic wall, and finally tying on the aortic side. For the destroyed right-coronary leaflet, a 21 neoleaflet of aortic wall was modeled using the template (Figure 2) and used to replace the leaflet (Video 1). The aortic intima was directed toward the ventricular side and the other leaflets. Patch size proved ample to precisely replace the missing right coronary leaflet, but if any question exists, it is prudent to select the larger size. Then during the procedure, the patch could be trimmed further or plicated. The neoleaflet was anchored at the central base with a 6-0 PROLENE mattress suture buttressed with fine Dacron pledgets. Interrupted simple 6-0 PROLENE sutures were used to suture the neoleaflet to the damaged leaflet rim and annulus until the commissures were reached. As a final step, Dacron-pledgetted 4-0 Tevdek mattress sutures were passed vertically through the top of the neoleaflet and to the outside of the aorta to secure the commissures. The FEL of the neoleaflet was trimmed to match the heights of the normal leaflets (Figure 3). The ascending aorta was replaced with a 24 mm Dacron graft.

### Clinical Outcome

After repair, the transesophageal echocardiogram showed good aortic valve leaflet motion, a vertical coaptation length of 8 to 9 mm, a mean gradient of 5 to 6 mm Hg, and no detectable AI (Figure 3; Video 1). The patient's recovery was uneventful. A transthoracic echocardiogram taken at 5 months postoperatively showed an ejection fraction of 67%, normal aortic valve function, and trace (grade 0) AI.

## Discussion

Early and late outcomes after prosthetic aortic valve replacement for IE have been poor.^1^^,^^2^^,^^E9^^,^^E10^ After valve replacement for IE, risk-adjusted early and late hazard ratios for mortality were 2 to 3 times those observed with standard etiologies. Operative mortalities have been suboptimal, and results have not been improving over time. Placement of a prosthetic valve as a large foreign body may predispose to reinfection, and all prostheses exhibit late valve-related complications. Although the patient subgroups were not strictly comparable, valve repair for IE has been associated with lower mortality,^1^ but limited applicability and late repair failure have been issues. Failure often has been attributable to degeneration of pericardium used to reconstruct leaflet defects. A better leaflet substitute would be useful.

For the aforementioned reasons, aortic wall patches could address many of these problems. Ideally, the infection should be fully treated and resolved preoperatively, as in the current case. If active infection persists, however, any dead or involved annular or aortic tissue should be debrided and reconstructed using glutaraldehyde-fixed bovine pericardium. If the annulus is 2 or more sizes larger than predicted by the leaflet ball sizer (#301, Corcym SRI), an appropriately sized annuloplasty ring (HAART 300, Corcym SRI) should be inserted to bring root and leaflet dimensions into a consistent 3-dimensional geometry.^E6^ As with mitral repair, a simple low-profile annuloplasty ring should not dramatically increase early or late reinfection rates. For acute IE, it might be better to replace harvested aortic tissue with something like a glutaraldehyde-fixed bovine pericardial tube, to reduce the chances of infecting a Dacron graft. In cases with severe leaflet defects, complete leaflet replacement using living aortic wall and precise template geometry (Figure 2) could ensure long-term stability. Use of aortic wall as pledgetted reinforcement of leaflet hole closures (as in the current case) or for reconstruction of commissural defects^E3^ also could reduce late repair failure. Finally, at the end, aortic wall leaflets could be plicated or managed with standard repair techniques,^E7^ as needed to fine-tune coaptation quality. Thus, increasing application of aortic wall leaflet reconstruction could improve results of IE surgery.

Long-term outcome data will be required for full validation of these concepts. To date, our collaborators have employed aortic wall for aortic valve repair in over 40 patients with a maximal follow-up of 3-years.^E4^ Clinical outcomes have been good, and echocardiographic patch morphology seems to remain constant over time, again suggesting stability of a living vascular tissue. Formal follow-up of these patients is planned in another year or two, which should provide sound data on which to base decisions. However at present, results seem good enough to proceed judiciously with this approach in carefully selected patients.

## Conflict of Interest Statement

Dr Rankin previously was a consultant for BioStable Science and Engineering. All other authors reported no conflicts of interest.

The *Journal* policy requires editors and reviewers to disclose conflicts of interest and to decline handling or reviewing manuscripts for which they may have a conflict of interest. The editors and reviewers of this article have no conflicts of interest.

## Supplementary Data

Video 1 Leaflet replacement for endocarditis using autologous aortic wall. Video available at: https://www.jtcvs.org/article/S2666-2507(25)00163-4/fulltext.
