# Supplementary figures and images for: Autologous leaflet reconstruction for aortic valve endocarditis
Source: JTCVS Tech. 2025 May 3;31:52–7. doi: 10.1016/j.xjtc.2025.04.016 (PMC12238035; doi:10.1016/j.xjtc.2025.04.016)

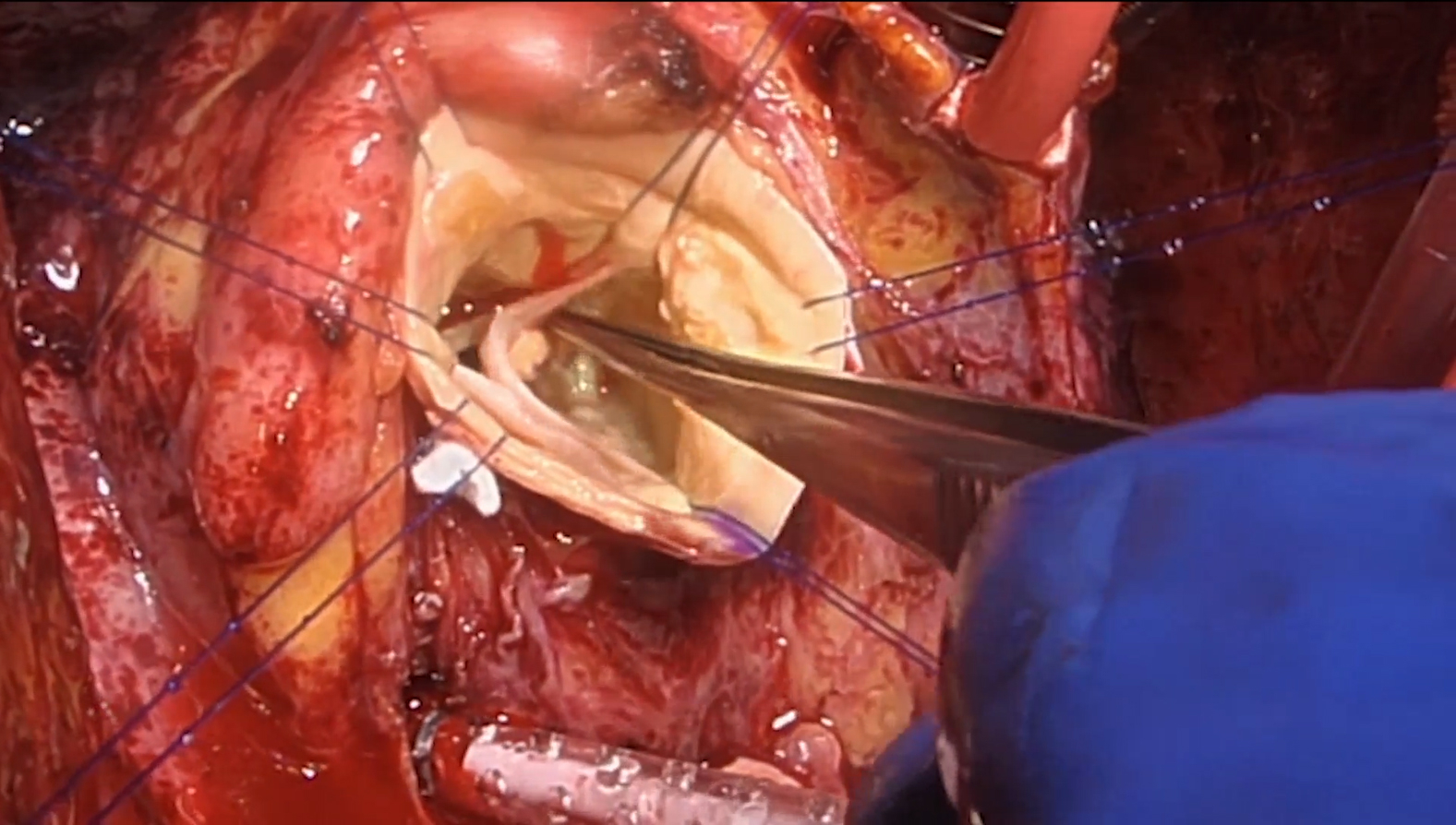

Supplement: Video 1 — Leaflet replacement for endocarditis using autologous aortic wall. Video available at: https://www.jtcvs.org/article/S2666-2507(25)00163-4/fulltext. [file fx2.jpg]
